# Appropriate empirical antifungal therapy is associated with a reduced mortality rate in intensive care unit patients with invasive fungal infection: A real-world retrospective study based on the MIMIC-IV database

**DOI:** 10.3389/fmed.2022.952611

**Published:** 2022-09-20

**Authors:** Man-ka Zhang, Zhi-guo Rao, Tao Ma, Ming Tang, Tian-qi Xu, Xiao-xu He, Zhou-ping Li, Yin Liu, Qing-jie Xu, Ke-yu Yang, Yi-fan Gong, Jing Xue, Mei-qing Wu, Xiao-yan Xue

**Affiliations:** Department of Critical Care Medicine, Aerospace Center Hospital, Beijing, China

**Keywords:** ICU, fungus, pre-emptive, antifungals, mortality, sepsis

## Abstract

**Objective:**

The study aimed to determine the prevalence and pathogens of invasive fungal infection (IFI) among intensive care unit (ICU) patients. The next goal was to investigate the association between empirical antifungal treatment and mortality in ICU patients.

**Methods:**

Using microbiological events, we identified all ICU patients with IFI and then retrieved electronic clinical data from the Medical Information Mart for Intensive Care IV (MIMIC-IV) database. The data were statistically analyzed using *t*-tests, chi-square tests, log-rank tests, and Cox regression.

**Results:**

The most commonly reported fungi were Candida (72.64%) and Aspergillus (19.08%). The most frequently prescribed antifungal medication was fluconazole (37.57%), followed by micafungin (26.47%). In the survival study of ICU patients and patients with sepsis, survivors were more likely to receive empirical antifungal treatment. In contrast, non-empirical antifungal therapy was significantly associated with poor survival in patients with positive blood cultures. We found that the current predictive score makes an accurate prediction of patients with fungal infections challenging.

**Conclusions:**

Our study demonstrated that empirical antifungal treatment is associated with decreased mortality in ICU patients. To avoid treatment delays, novel diagnostic techniques should be implemented in the clinic. Until such tests are available, appropriate empirical antifungal therapy could be administered based on a model that predicts the optimal time to initiate antifungal therapy. Additional studies should be conducted to establish more accurate predictive models in the future.

## Introduction

Fungal pathogens can cause invasive fungal infections (IFI), complex chronic respiratory disorders, and severe chronic diseases. Additionally, these pathogens can also cause recurrent infections. According to earlier studies (1, 2), IFI in the intensive care unit (ICU) is associated with high morbidity and mortality.

Existing studies have evaluated whether patients with Candida infection benefit from empirical antifungal therapy (3–11). These investigations either did not incorporate all fungal pathogens and infectious sites or did not involve a substantial sample size. Empirical antifungal therapy in the ICU is a complex issue, and few clinical studies have assessed its efficacy (12). Moreover, in patients with sepsis, some observational studies have shown that empirical antifungal therapy may be associated with a reduction in mortality. However, these studies have not confirmed the causal relationship between antifungal therapy and prognosis (13–15). Furthermore, Hoenigl et al. (16) and Niederman et al. (17) attributed the emergence of antifungal resistance and antimicrobial overuse to the increased use of empirical therapy. The severe consequences of fungus among ICU patients were apparent in our study, with a crude mortality rate of 20%. There is an urgent need to identify the possible determinants of mortality in ICU patients to improve this poor prognosis. Some studies (4, 6) found a correlation between empirical antifungal therapy and ICU patient mortality, whereas others (5, 7) did not. Therefore, we aimed to determine if empirical antifungal therapy was associated with lower mortality among ICU patients with fungal infections.

Our research was a retrospective cohort study with two primary objectives. First, we aimed to identify the pathogens and medications used in patients with IFI in the ICU to mitigate the impact on mortality risk due to the selection of a relatively narrow antibacterial spectrum. Our next goal was to determine whether there was an association between empirical antifungal treatment and increased mortality risk in ICU patients.

## Materials and methods

### Data source

All patients were screened from the Medical Information Mart for Intensive Care IV (MIMIC-IV version 0.4) database from 2008 to 2019 (18, 19). The institutional review boards at the Massachusetts Institute of Technology and Beth Israel Deaconess Medical Center authorized the investigator access to the database for research purposes after completing the online course “Protecting Human Study Participants” (20). Man-ka Zhang, the first author, completed the Collaborative Institutional Training Initiative examination (certification number: 35842360) and accessed the database.

### Patient population

From 2008 to 2019, the total number of patients in the MIMIC-IV database was 257,366, of which 50,048 were admitted to the ICU. Only patients with IFI were included in this study. We characterized IFI as the positive culture for fungal species in the deep site. The inclusion criteria were as follows: (1) adults aged between 18 and 89 years; and (2) the presence of microbiological events positive for fungal species in a deep site for the first time. Due to the high contamination rate of urine samples, we only included patients with a positive urine culture from renal pelvis puncture and suprapubic sampling. The exclusion criteria were: (1) patients with limited clinical data (21); and (2) Candida identification in respiratory specimens; we only analyzed the hospitalization data of patients who reported fungal positivity for the first time.

### Evaluating the outcome

The primary outcome was mortality from any cause. The information extracted in the study included clinical characteristics, laboratory results, comorbidities, severity scoring system, and treatment. The following patient-specific hospitalization information was collected:

(1) Characteristics: age, gender, time of admission, time of discharge, and time of death.

(2) Comorbidities: hematological malignancy, sepsis, congestive heart failure, myocardial infarction, cerebrovascular disease, peripheral vascular disease, dementia, rheumatic disease, chronic pulmonary disease, peptic ulcer disease, mild liver disease, diabetes, paraplegia, renal disease, cancer, severe liver disease, metastatic solid tumor, and AIDS.

(3) Laboratory data: microbiological data and positive reporting time.

(4) Severity scoring system: the Acute Physiology and Chronic Health Evaluation (APACHE) III and the Charlson comorbidity index.

(5) Treatment: antifungal drugs, broad-spectrum antibiotics, abdominal surgery, parenteral nutrition, central venous catheterization, ventilation, and hemofiltration.

### Data analysis

Data processing was performed using Python 3.8, which is an interpreted high-level, general-purpose programming language. Baseline characteristics of hospital survivors and non-survivors in patients with IFI were compared using the chi-square test for categorical variables and Student’s *t*-test for normally distributed continuous variables. Survival over the 28-day period was compared using Kaplan–Meier curves and log-rank testing. Mortality over the 28-day interval was predicted using multivariate Cox regression analyses. The Cox regression analysis included factors that supported the Proportional hazards (PHs) hypothesis. Statistical analyses were performed using the Statistical Product Service Solutions (SPSS) for Windows (SPSS 26, Inc., Chicago, IL, United States). Forest plots were constructed using GraphPad Prism version 9.0.0. (GraphPadSoftware, Inc., La Jolla, CA). All tests were two-tailed, with a *p*-value < 0.05 considered statistically significant.

### Scoring system

Appropriate initiation of antifungal therapy is frequently delayed because of the low sensitivity of microbial cultures and the time required for cultures to become positive. Several studies have attempted to identify empirical treatment strategies based on the risk factors to address this issue. We selected a scoring system for the early prediction of IFI that could potentially be applied to our cohort for validation. The scoring system included seven significant risk factors: gastrointestinal surgery (5 points), diabetes mellitus (5 points), hematological malignancies (4 points), the use of broad-spectrum antibiotics for ≥ 4 days (4 points), a central venous catheter (3 points), total parenteral nutrition (3 points), and mechanical ventilation for ≥ 2 days (2 points). The risk score has a theoretical range of 0–26. A higher score suggests a higher risk of infection. Based on these results, three discrete risk-level categories were established: low (score ≤ 8), intermediate (score 9–13), and high (score ≥ 14) (22).

## Results

### Demographics and clinical features of patients with invasive fungal infection in intensive care unit

In total, 1,981 patients with IFI were included in our study. All data were anonymized. The age range was 18–89 years, with a mean age of 59.61 ± 15.37 years. There were 1,075 (54.27%) men and 906 (45.73%) women. The mean APACHE III score was 72.87 ± 30.58 (range: 10–178). The mean Charlson comorbidity index was 5.86 ± 3.10 (range: 0–18). 397 patients (20.04%) died during hospitalization. Non-survivors were significantly older, had higher APACHE III scores and Charlson comorbidity index, were less likely to receive empirical antifungal treatment, and needed ventilator and hemofiltration treatment (Table 1).

**TABLE 1:** Demographics and clinical features.

Characteristics	Hospital survivors (*n* = 1584 79.96%)	Hospital non-survivors (*n* = 397 20.04%)	*P*-value
Age (year)	58.71 ± 15.45	63.20 ± 14.51	<0.001
**Gender [no. (%)]**			
Male	865 (43.67)	210 (10.60)	0.540
Female	719 (36.29)	187 (9.44)	
APACHE III score	63.19 ± 27.62	91.73 ± 28.16	<0.001
Charlson_ comorbidity_index	5.56 ± 3.06	7.04 ± 2.97	<0.001
**Antifungal treatment [no. (%)]**			
Empirical antifungal treatment	988 (49.87)	279 (14.08)	0.003
Non-empirical antifungal treatment	596 (30.09)	118 (5.96)	
**Mechanical ventilation [no. (%)]**			
Yes	720 (36.35)	353 (17.82)	<0.001
No	864 (43.61)	44 (2.22)	
**Renal replacement therapy [no. (%)]**			
Yes	158 (7.98)	145 (7.32)	<0.001
No	1,426 (71.98)	252 (12.72)	

### Microbiological data of patients with invasive fungal infection in intensive care unit

**FIGURE 1:**
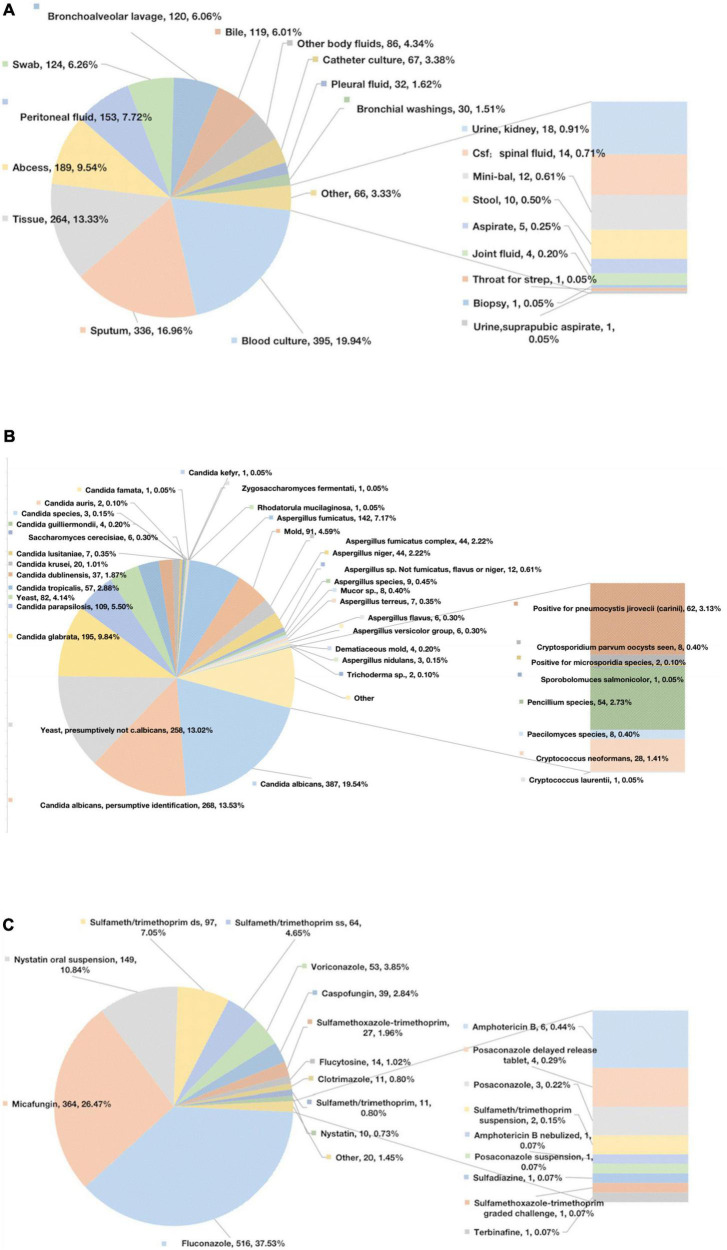
Microbiological data. **(A)** The sites of infection of fungus. **(B)** Specific positive fungal species. **(C)** Specific antifungal drugs.

In our study, the bloodstream was the most common site of fungal infection, accounting for 19.94% of infections, followed by sputum (16.96%), tissue (13.33%), and abscesses (9.54%) (Figure 1A). The most commonly reported fungal species were *Candida* spp. (72.64%) and *Aspergillus* spp. (19.08%) (Figure 1B). In our study, the most frequently prescribed antifungal drugs were fluconazole (37.53%) and micafungin (26.47%) (Figure 1C).

### Survival analysis of patients with invasive fungal infection in intensive care unit

**FIGURE 2:**
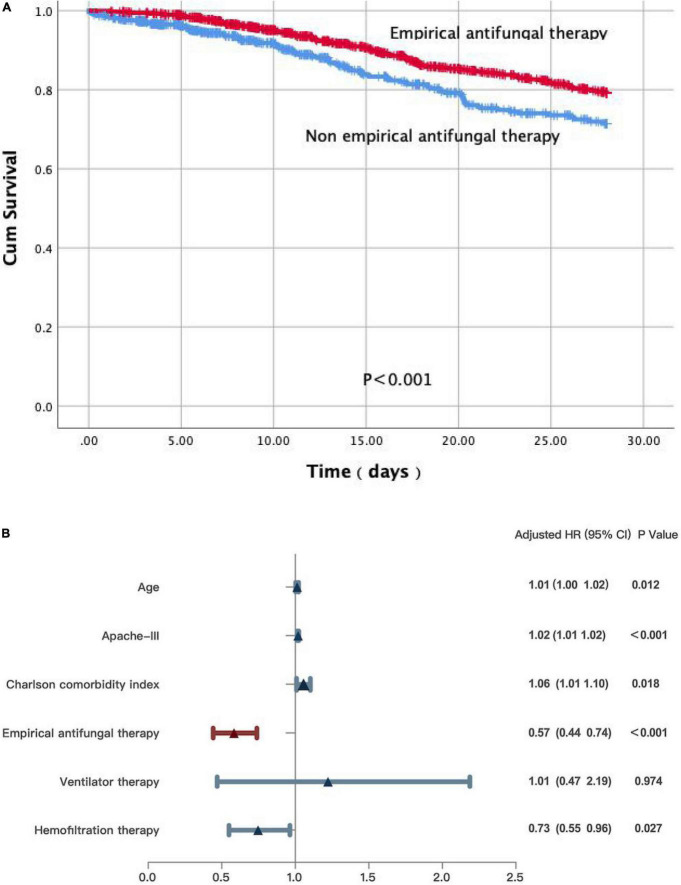
**(A)** A Kaplan-Meier survival analysis and a log-rank test on patients with IFI in ICU. **(B)** Multivariate Cox regression analyses for patients with IFI in ICU.

We further performed the Cox regression and log-rank analyses for the primary outcomes of the 28-day mortality. Non-empirical antifungal treatment (*p* < 0.001) was significantly associated with decreased survival (Figure 2A). According to the Cox PHs model, non-survivors were more likely to be older [hazard ratio (*HR*) 1.01; 95% *CI*: 1.00–1.02; *p* = 0.012], have higher APACHE III scores (*HR* 1.02; 95% *CI*: 1.01–1.02; *p* < 0.001), or have a higher Charlson comorbidity index (*HR* 1.06; 95% *CI*: 1.01–1.10; *p* = 0.018) compared with that of survivors. Survivors were more likely to receive empirical antifungal treatment (*HR* 0.57; 95% *CI*: 0.44–0.74; *p* < 0.001), and hemofiltration therapy (*HR*, 0.73; 95% *CI*: 0.55–0.96; *p* = 0.027). Ventilator therapy (*HR*, 1.01; 95% *CI*: 0.47–2.19; *p* = 0.974) did not differ significantly between the survivors and non-survivors (Figure 2B).

**FIGURE 3:**
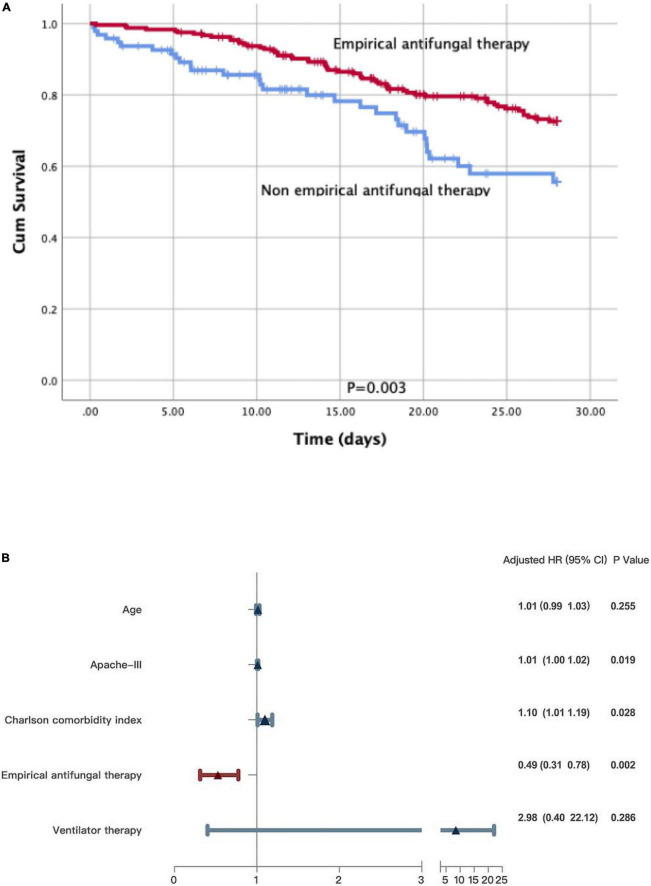
**(A)** A Kaplan-Meier survival analysis and a log-rank test on patients with sepsis. **(B)** Multivariate Cox regression analyses for patients with sepsis.

The Kaplan–Meier survival analysis and the log-rank test revealed that non-empirical antifungal therapy (*p* = 0.003) was significantly associated with poor survival in a subgroup of patients with sepsis (Figure 3A). Significant differences were detected in the APACHE III scores (*HR* 1.01; 95% *CI*: 1.00–1.02; *p* = 0.019), the Charlson comorbidity index (*HR* 1.10; 95% *CI*: 1.01–1.19; *p* = 0.028), and empirical antifungal treatment (*HR* 0.49; 95% *CI*: 0.31–0.78; *p* = 0.002). There was no statistically significant difference in the use of ventilation therapy (*HR* 2.98; 95% *CI*: 0.40–22.12; *p* = 0.286) or age (*HR* 1.01; 95% *CI*: 0.99–1.03; *p* = 0.255) between the survivors and non-survivors (Figure 3B).

**FIGURE 4:**
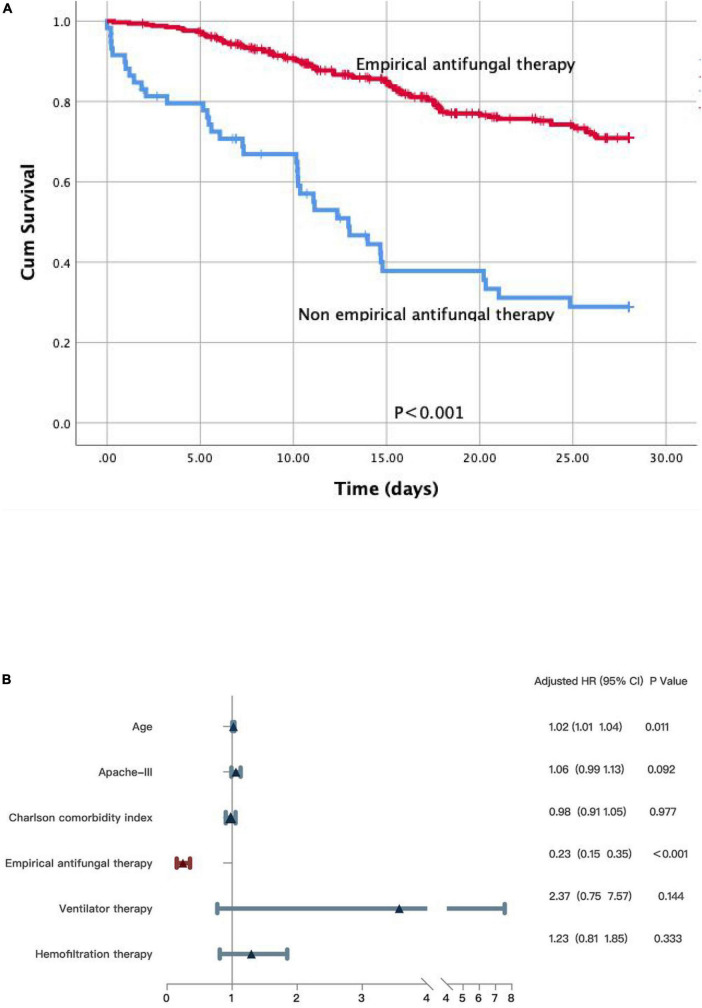
**(A)** A Kaplan-Meier survival analysis and a log-rank test on the subgroup patients with fungus positive in blood culture. **(B)** Multivariate Cox regression analyses for patients with fungus positive in blood culture.

We reached the same conclusion in patients with positive blood culture results. We further performed the Kaplan–Meier survival analysis and the log-rank test on the subgroup of patients with fungus-positive blood cultures. Non-empirical antifungal medication (*p* < 0.001) was significantly related to poor survival (Figure 4A). Significant differences were detected in age (*HR* 1.02; 95% *CI*: 1.01–1.04; *p* = 0.011), the APACHE III scores (*HR* 1.06; 95% *CI*: 0.99–1.13; *p* = 0.092), the Charlson comorbidity index (*HR* 0.98; 95% *CI*: 0.91–1.05; *p* = 0.977), and empirical antifungal treatment (*HR* 0.23; 95% *CI*: 0.15–0.35; *p* < 0.001). Meanwhile, survivors and non-survivors did not significantly differ in their use of ventilation (*HR* 2.37; 95% *CI*: 0.75–7.57; *p* = 0.144) and hemofiltration therapy (*HR* 1.23; 95% *CI*: 0.81–1.85; *p* = 0.333) (Figure 4B).

### Validation of the existing scoring system for patients with invasive fungal infection in intensive care unit

**FIGURE 5:**
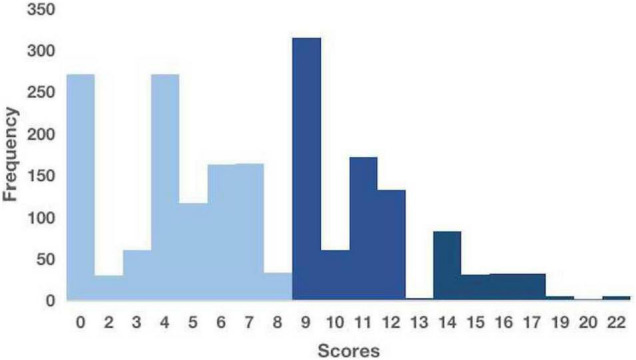
Distribution of scores for our patients.

Due to the limitations of retrospective studies, we could only obtain information on patients’ “parenteral nutrition,” not “total parenteral nutrition.” Thus, the overall score may be inflated relative to the actual scenario. In our cohort, participants at low, intermediate, and high risks of developing IFI were 55.93%, 34.48%, and 9.58%, respectively (Figure 5).

## Discussion

Our study revealed an association between empirical antifungal medication and reduced mortality in a large sample size of ICU patients with IFI. In addition, fungus-induced sepsis and septic shock were particularly prevalent in the ICU and were directly associated with poor prognosis (23–26), as indicated in the analysis of the subgroup of patients with sepsis. Finally, patients with positive fungal blood cultures who received non-empirical antifungal therapy had significantly lower chances of survival.

We then comprehensively analyzed the pathogens involved and the treatments used in the vast cohort of adult ICU patients. The blood and sputum samples had the highest fungal positivity rates. Therefore, raising awareness of the importance of sending samples for analysis is beneficial to identify the fungus at an early stage. The fungi with the highest detection rate in the ICU were *Candida* spp. and *Aspergillus* spp. (27). During the early stages, fluconazole and micafungin were the most commonly prescribed antifungal medicines. Micafungin is an echinocandin antifungal drug with action against *Candida* spp. and *Aspergillus* spp. (28–30). High-quality clinical trials are needed to establish that echinocandins may be an attractive alternative to fluconazole for primary IFI prophylaxis, considering their broader antimicrobial spectrum, lower toxicity, and decreased incidence of drug-to-drug interactions in humans.

Using multivariate analysis, Morrell et al. demonstrated that the administration of antifungal medication more than 12 h after the initial positive culture was associated with hospital mortality. This finding emphasizes the clinical significance of early and proper treatment of patients with fungal bloodstream infections (6). Meanwhile, Marriott et al. proposed that failure to initiate appropriate empirical antifungal therapy may be a modifiable risk factor for mortality (14). However, Kollef et al. found no connection between in-hospital mortality and early initiation of antifungal therapy (10). Peçanha-Pietrobom et al. (31) suggested that neither antifungal prophylaxis nor empirical therapy reduces invasive candidiasis-related mortality in critical care settings (32–34). Our study indicates that the non-empirical antifungal treatment is associated with higher mortality in ICU patients. Consequently, the early detection of fungal infections could drastically reduce mortality rates. The lack of specific clinical findings suggests that the diagnosis is a concern in impeding the early detection and treatment of fungal infections. Next generation sequencing (NGS) is a high-throughput, large-scale parallel sequencing technique that can simultaneously sequence thousands to billions of DNA fragments independently (35). If NGS becomes more prevalent in clinical settings, it may achieve the goal of early detection of fungal infections. However, its high cost remains an obstacle to its widespread clinical implementation. Meanwhile, many researchers have proposed the use of clinical prediction models to identify patients with high-risk factors for fungal infection (36–38). However, due to their complexity, most of the existing prediction models for patients with IFI in the ICU are challenging to promote and implement in clinical practice. We selected a simple and feasible clinical prediction model for validation; however, it was challenging to accurately identify patients at high risk of IFI in our cohort. Therefore, a faster and more accurate diagnosis of fungal infections may be the most effective method to avoid treatment delays. Future clinical researchers need to evaluate the operational aspects of these techniques and assess whether molecular diagnostics can be developed for cost-effective and efficient usage in clinical laboratory settings (6).

Our study had several limitations. First, since this was a real-world retrospective study, we could not account for all confounding factors that may exist in patients with IFI. Similar to previous reports, its retrospective heterogeneous characteristics were the primary factors to be considered as insufficiencies, owing to the low sensitivity of fungal infection culture and the difficulties of clinical diagnosis. Since we were examining the relationship between empirical treatment and clinical outcomes, a retrospective study can raise uncertainty regarding timeliness. Simultaneously, the actual clinical context of specific drug usage decisions at that time was insufficient. All of these factors affected the outcomes of this investigation. Second, we did not assess the drug susceptibility data of the identified pathogens, and crucial information regarding the susceptibility of the fungus was lacking. Therefore, we could not evaluate the effect of inappropriate antifungal therapy on patient mortality and the influence of patients’ susceptibility to the fungus on the choice of empiric treatment. However, these were not the primary objectives of our investigation. Third, the current findings were based on a single center and need to be validated in other settings. Fourth, due to the limitation of clinical data in the database, patients older than 89 years were consistently recorded as 89 years old in the MIMIC database; therefore, we excluded patients older than 89 years for the fear of influencing statistical results. Only 28-day mortality was analyzed because there were too many missing clinical data points to obtain high-quality statistical results. Finally, the multivariate analysis revealed a statistically significant relationship between empirical antifungal therapy and outcomes in ICU patients. This highlights the complexity of the variables that can influence patient outcomes in severe infections.

## Conclusions

Our study demonstrates that empirical antifungal therapy is associated with a decreased mortality rate in ICU patients. Novel diagnostic techniques should be implemented in the clinic to prevent delayed treatment. In the absence of such testing, appropriate empirical antifungal therapy may be administered based on a prediction model indicating when to initiate empirical antifungal therapy. Finally, additional studies should be conducted to develop more accurate predictive models in the future.

## Data availability statement

The original contributions presented in this study are included in the article/supplementary material, further inquiries can be directed to the corresponding author.

## Ethics statement

The studies involving human participants were reviewed and approved by the Institutional Review Board at the Massachusetts Institute of Technology and Beth Israel Deaconess Medical Center granted the investigator access to the database for research after completing the online course “Protecting Human Study Participants”. Written informed consent for participation was not required for this study in accordance with the national legislation and the institutional requirements.

## Author contributions

X-YX and M-KZ led the team and were responsible for all aspects of the research. Z-GR, TM, MT, T-QX, and X-XH contributed to the methods, results, and interpretation. Z-PL, YL, Q-JX, and K-YY participated in designing and writing the manuscript. Y-FG, M-QW, and JX revised the manuscript. All authors contributed to the article and approved the submitted version.
